# Prognostic Relevance of Nonsustained Ventricular Tachycardia in Patients with Pulmonary Hypertension

**DOI:** 10.1155/2016/1327265

**Published:** 2016-12-20

**Authors:** Dirk Bandorski, Harilaos Bogossian, Johanna Stempfl, Werner Seeger, Matthias Hecker, Ardeschir Ghofrani, Reinhard Hoeltgen, Henning Gall

**Affiliations:** ^1^University of Giessen and Marburg Lung Center (UGMLC), The German Center for Lung Research (DZL), Klinikstrasse 33, 35392 Giessen, Germany; ^2^Klinikum Lüdenscheid, Universität Witten-Herdecke, Medizinische Klinik III, Paulmannhöherstr. 14, 58515 Lüdenscheid, Germany; ^3^Klinikum Westmünsterland, St. Agnes-Hospital Bocholt-Rhede, Medical Clinic 1-Cardiology/Electrophysiology, Barloer Weg 125, 46397 Bocholt, Germany

## Abstract

*Background.* Increased pulmonary vascular resistance in patients with pulmonary hypertension (PH) leads to an increased afterload of right heart and cardiac remodeling which could provide the substrate or trigger for arrhythmias. Supraventricular arrhythmias were associated with clinical deterioration but were not associated with sudden cardiac death (SCD). SCD has been reported to account for approximately 30% of deaths in patients with pulmonary arterial hypertension (PAH).* Objective.* The role of nonsustained ventricular tachycardia (nsVT) and its prognostic relevance in patients with PH remains unclear. This study evaluated the prognostic relevance of nsVT in patients with PAH and chronic thromboembolic pulmonary hypertension (CTEPH).* Methods.* Retrospectively, patients with PAH and CTEPH who underwent Holter ECG monitoring and available data of survival were investigated.* Results.* Seventy-eight (PAH: 55, CTEPH: 23) patients were evaluated. Holter ECG revealed nsVT in 12 patients. Twenty-one patients died during follow-up. In patients with nsVT, tricuspid annular plane systolic excursion was lower (*p* = 0.001), and systolic pulmonary arterial pressure was higher (*p* = 0.163). Mean survival of patients without/with nsVT was 155.2 ± 8.5/146.4 ± 21.4 months (*p* = 0.690). The association between arrhythmias and survival was not confounded by age (*p* = 0.681), gender (*p* = 0.752), 6-MW distance (*p* = 0.196), or arterial hypertension (*p* = 0.238).* Conclusions.* In patients with PH, nsVT occurs more often than previously reported, and patients with PH group 1 seem to be more at risk.

## 1. Introduction

Pulmonary arterial hypertension (PAH) and chronic thromboembolic pulmonary hypertension (CTEPH) are characterized by progressive obliteration of precapillary pulmonary vessels leading to an increased pulmonary vascular resistance (PVR). The subsequent increase in PVR leads to an increased afterload of the right heart and cardiac remodeling [1]. Elevated right heart filling pressures and cardiac remodeling could provide the substrate or trigger for arrhythmias [2, 3]. Five studies evaluated the incidence of supraventricular arrhythmias in patients with PH, which varied between 11.7% and 25.1% over periods of 6–13 years [4–8]. Supraventricular arrhythmias were associated with clinical deterioration but were not associated with sudden cardiac death (SCD) [4–8]. SCD has been reported to account for approximately 30% of deaths in patients with PAH [9]. In our own data, 13% of the PH patients showed nonsustained ventricular tachycardia (nsVT) during Holter ECG monitoring [10]. Because of missing follow-ups in the study, the role of nonsustained ventricular tachycardia and its prognostic relevance in patients with PH remains unclear. The recent study was performed to evaluate the prognostic relevance of nsVT in patients with PAH and CTEPH.

## 2. Methods

Retrospectively, patients with PAH and CTEPH from two previous studies [10, 11] who underwent Holter ECG monitoring (University of Giessen, Medical Clinic I and Medical Clinic 2 and Kerckhoff-Klinik, Department of Cardiology) and available data of survival on 2015.06.30 were investigated. To match our results with other studies [4–6, 8], we investigated the survival rate in patients of PH groups 1 and 4. Data collection included demographic data, etiology of PH, comorbidities, survival, cause of death, and data from echocardiography. Hemodynamic data from right heart catheterization were included if performed six months prior to Holter ECG monitoring. The study and the study design were approved by the institutional review board (reference number: 184/15).

## 3. Holter ECG Monitoring

For assessment of arrhythmias, all patients underwent Holter ECG recording (GETEMED, Teltow, Germany) for 24 [11] or 72 hours [10]. The recordings were analyzed by an operator-controlled analysis (GETEMED, Teltow, Germany). Classification of documented nsVT (three or more beats in duration, terminating spontaneously in less than 30 s) was performed in accordance with the terminology of the American Society of Cardiology, American Heart Association, and European Society of Cardiology [12]. The results of the Holter ECG recordings were correlated with six-minute walk distance and data of echocardiography and right heart catheterization.

## 4. Six-Minute Walk (6-MW) Testing 

Patients walked along a 100-foot floor at their own pace to cover as much distance as possible. The total accessible distance was determined. The tests were performed in accordance with the guidelines of the American Thoracic Society (ATS) [13].

## 5. Echocardiography

Transthoracic echocardiographic studies were performed by experienced investigators (GE, Connecticut, USA). Collected data contained right atrial (RA) diameter (four-chamber view, measured at the end systole), right ventricle (RV) diameter (four-chamber view, measured at the end diastole), left ventricular ejection fraction (LV-EF, biplane Simpson method, two- and four-chamber view), tricuspid annular plane systolic excursion (TAPSE), and systolic pulmonary artery pressure (sPAP). All measurements were carried out in accordance with the guidelines of the American Society of Echocardiography [14–17].

## 6. Right Heart Catheterization

Right heart catheterization was performed via the right jugular vein. The hemodynamic measurements included mean right atrial pressure (RAP), mean pulmonary pressure (mPAP), pulmonary capillary wedge pressure (PCWP), pulmonary vascular resistance (PVR), and cardiac index (CI, thermodilution or Fick method, as appropriate).

## 7. Statistical Analysis

Statistical calculations were performed using SPSS (ver. 21; IBM, Armonk, NY). Patient data are presented as absolute numbers (mean or median) and standard deviations (SDs). Comparisons between groups were done using* t*-test or chi-square test as appropriate.* p* values of <0.05 were considered statistically significant.

Cox regression analyses were performed to evaluate the influence of nsVT on survival. Multivariate Cox regression was used to adjust for possible confounders, namely, age, six-minute walk distance (6-MWD), echocardiographic parameters (sPAP, diameter of RA/RV, TAPSE, and LV-EF), and hemodynamic parameters (RAP, PVR, PCWP, mPAP, CI, and ven sO2).

Kaplan-Meier plots were used to illustrate survival in relation to ventricular tachycardia. Statistical analyses were performed using data obtained during the entire observation period (up to 200 months).

## 8. Results

A total of 78 patients were evaluated: 55 with PAH and 23 with CTEPH (7 patients suffered from persistent CTEPH after pulmonary artery embolectomy and 16 patients declined pulmonary embolectomy). Holter ECG revealed nsVT in 12 patients (PAH: 8 patients; CTEPH: 4 patients). Patients walked 370 ± 125 m in six minutes (patients without nsVT: 387 ± 281 m; patients with nsVT: 283 ± 109; *p* = 0.941). General characteristics are presented in Table 1.

Mean time from first diagnosis of PH to Holter ECG was 5.4 ± 3.9 years for patients without nsVT and 6.2 ± 4.1 years for patients with nsVT (*p* = 0.736). In patients with PAH, the mean interval between first diagnosis of PH and Holter ECG was 5.5 ± 4.1 years and 5.7 ± 3.7 years in patients with CTEPH (*p* = 0.496). Twenty-one patients [26.9%, 17 patients without nsVT (PH group 1 + 4) and 4 patients with nsVT (PH group 1)] died during follow-up, caused by right heart failure in 7 patients, sudden cardiac death in 6 patients, respiratory insufficiency in 2 patients, and decompensated aortic valve stenosis in 1 patient, and in 5 patients the cause of death was unknown. In the subgroup of patients with nsVT, causes of death were right heart failure in 2 patients, sudden cardiac death in 1 patient, and decompensated aortic valve stenosis in 1 patient. The mean interval from first diagnosis to death was 8.4 ± 3.9 years in patients without nsVT and 7.9 ± 3.5 years in patients with nsVT (*p* = 0.185). The average interval from first diagnosis to death was 8.2 ± 3.9 years in patients with PAH and 8.5 ± 3.8 years in patients with CTEPH (*p* = 0.855).

## 9. ECG Parameters 

Neither in patients with or without arrhythmia nor between survivors' and nonsurvivors' heart rate did mean QRS width, QT interval, and corrected QT interval (QTc) show any statistical significance. Mean QTc in patients with nsVT who died during follow-up was 445 ms (data not shown).

## 10. Echocardiography and Right Heart Catheterization 

Cardiac function and hemodynamic parameters were evaluated by echocardiography and right heart catheterization. Echocardiography was performed in all patients, and data of right heart catheterization within the last 6 months before Holter ECG monitoring were available in 49 patients (40/66 patients without nsVT and 9/12 patients with nsVT). TAPSE was significantly lower in patients with nsVT (*p* = 0.001), and sPAP was higher in patients with nsVT (*p* = 0.163). Right heart catheterization revealed higher mPAP (*p* = 0.865) and PVR (*p* = 0.353) and lower CI (*p* = 0.390) in patients with nsVT. Echocardiography showed normal LV-EF in 77 patients and low reduction in one patient (no coronary heart disease, TAPSE 14 mm) with nsVT and CTEPH (not shown in Table 2). Diameter of the right atrium was larger (48.3 versus 43 mm; *p* = 0.013), TAPSE was lower (13.7 versus 16.7 mm; *p* = 0.005), and CI was similar (2.57 versus 2.41 l/min/m^2^; *p* = 0.010) in patients with nsVT who died during follow-up (data not shown).

## 11. Survival and Arrhythmias

**Figure 1:**
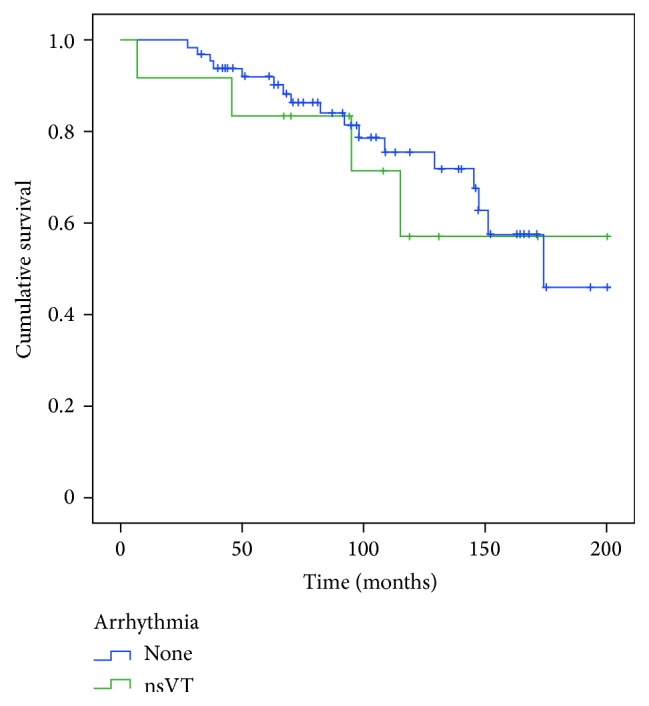
Nonsustained ventricular tachycardia and survival. nsVT: nonsustained ventricular tachycardia.

Mean survival of patients without/with nsVT (Figure 1) was 155.2 ± 8.5/146.4 ± 21.4 months without statistical significance (*p* = 0.690). Multivariate analysis revealed that the association between arrhythmias and survival was not confounded by age (*p* = 0.681), gender (*p* = 0.752), 6-MW distance (*p* = 0.196), or arterial hypertension (*p* = 0.238).

## 12. Discussion 

Investigations analyzing the prognostic relevance of nsVT in patients with pulmonary hypertension are lacking. Only three studies report on ventricular arrhythmia during 24–72-hour Holter ECG [10, 11, 18], but none of these studies report on the prognostic relevance of nonsustained VT. We retrospectively analyzed our data to discover the prognostic relevance of nsVT, as none of the mentioned studies revealed any respective information. To match our results with other studies [4–6, 8], we investigated survival in patients (*n* = 78) of PH groups 1 and 4. Our data showed no significant difference of survival in patients with and without nsVT (7.9 ± 3.5 years versus 8.4 ± 3.9 years; *p* = 0.185). Mean times from diagnosis to Holter ECG were similar for both groups (with/without nsVT: 6.2 ± 4.1 years versus 5.4 ± 3.9 years; *p* = 0.736), indicating that the duration of PH does not seem to influence the probability of nsVT.

Nonsustained VT can be recorded in a wide range of different conditions, from apparently healthy individuals to patients with significant left heart disease. Its prognostic significance varies, depending on the underlying conditions (ischemic heart disease, cardiomyopathies, etc.) [19]. Depending on underlying diseases, antiarrhythmic therapy is recommended [19]. In contrast to patients with left heart disease, ventricular arrhythmias in patients with pulmonary hypertension are reported to be rare. The prognostic relevance of nsVT in patients with PH remains unclear. No trials to investigate the indications for antiarrhythmic therapy have been conducted in patients with PAH, and the ACC/AHA/ESC guidelines do not recommend prophylactic antiarrhythmic therapy in patients with PH either [12]. Two studies report sudden cardiac death (SCD) as the reason for about 30% of deaths in patients with pulmonary arterial hypertension, and SCD appears more common in patients with primary PAH than in those with CTEPH [20, 21]. In Hoeper's study, reporting the outcome after cardiopulmonary resuscitation, initial ECG presented ventricular fibrillation in only 8% of their patients. The other cases shared bradycardia (45%), electromechanical dissociation (28%), asystole (15%), or other arrhythmias (4%) [21].

Umar et al. evaluated the incidence of SCD in rats with PH induced by subcutaneous injection of monocrotaline [3]. Right ventricular ejection fraction deteriorated between the 14th day and 21st day after injection from 72 to 38%. The highest incidence of SCD occurred between days 23 and 32. Early afterdepolarizations (EADs) from the right ventricular epicardial surface triggered VT, which degenerated to ventricular fibrillation [3]. Two underlying mechanisms maintaining ventricular arrhythmias were identified by the authors: focal and incomplete reentrant wave fronts during ventricular fibrillation. The drop of the RV-EF occurred 10 days before the rise of SCD in the MCT-treated rats, and therefore the authors concluded that electric failure rather than hemodynamic deterioration was the reason for ventricular fibrillation. It is well known that EADs in humans can trigger malignant arrhythmias as torsade de pointes [22]. Theoretical and experimental studies identified reduced repolarization reserve, increased myocardial fibrosis, and elevated cytosolic Ca levels that promote EADs as mechanisms triggering tachyarrhythmias [23–28]. Cardiac MRI revealing delayed contrast enhancement [29, 30] and newly technical innovations like myocardial T1 mapping [31] might be helpful in identifying patients with cardiac fibrosis.

Echocardiographic and hemodynamic data such as sPAP, TAPSE, PVR, mPAP, CI, ven sO_2_, and RA/RV diameter were in accordance with the studies of Olsson et al. [4] and Wen et al. [8], investigating PH patients (PAH and CTEPH) with and without supraventricular arrhythmias. The studies revealed elevated sPAP, mPAP, and RA/RV diameters and reduced CI, ven sO_2_, and TAPSE in patients with supraventricular arrhythmias [4, 8]. The prognostic value of TAPSE was evaluated in three studies by Forfia et al. and Ghio et al. [32–34]. The study of Forfia et al. revealed that the survival of patients with a TAPSE less than 1.8 was significantly shorter than that of patients with a TAPSE of 1.8 cm or greater [32]. In Ghio's first study, TAPSE < 15 mm was strictly related to prognosis, and patients with TAPSE ≤ 15 mm had a nearly threefold event rate compared to patients with TAPSE of 15 mm (HR = 2.74) [33]. The second study of Ghio et al. confirmed these results [34]. A TAPSE ≥ 15 mm at baseline/after therapy was associated with a significantly lower risk of death or clinical worsening [34].

Values of mPAP, sPAP, CI, PVR, and ven sO_2_ in these three studies (if recorded in the study) were similar to our patients with nsVT, whereas RAP (8.5/10 mmHg versus 4 mmHg), PCWP (10 mmHg versus 7.9 mmHg), diameter of RA (48 mm versus 46 mm), and diameter of RV (46 mm versus 42 mm) were lower in our patients.

In summary, in patients with nsVT during follow-up, hemodynamic parameters (mPAP, sPAP, CI, PVR, and ven sO_2_) were similar to patients with supraventricular arrhythmias, whereas RAP was lower.

The prognostic value of QRS width and QT interval/corrected QT interval was analyzed in three studies [35–37]. Sun et al. analyzed QRS width in the initial 12-lead ECG in 212 patients with idiopathic PH [35]. The study revealed an increase of QRS width ≥ 0.12 seconds, observed in 16.5% of patients, as an independent predictor of mortality, being associated with a 2.5 times higher risk of death. QRS prolongation was positively correlated with right atrium and right ventricle diameters, indicating that right ventricular overload may play a role in the pathogenesis of PH. Hemodynamic variables were similar in the two groups (gp 1: QRS < 120 ms and gp 2: QRS ≥ 120 ms): mRAP (gp 1: 7.4 mmHg ± 5.9; gp 2: 8.6 mmHg ± 7.8; *p* = 0.30), mPAP (gp 1: 61.1 mmHg ± 16.1; gp 2: 60.9 mmHg ± 18.7; *p* = 0.53), and PVR (gp 1: 17 Wood units ± 10.3; gp 2: 18.5 Wood units ± 9.8; *p* = 0.69). In our study, values (except ven sO_2_) of hemodynamic parameters were even lower than those of patients in group 1. RA diameter (gp 1: 59.6 mm ± 11.0; gp 2: 77.4 mm ± 15.6) was lower and RV diameter was higher (gp 1: 31.4 mm ± 8.5; gp 2: 38.6 mm ± 9.4) than in our patients. Only 4 of our patients had a QRS width > 120 ms.

Two studies compared the duration of the QTc intervals in patients with different types of PH and in controls [36, 37]. In Hong-Liang's study, the QTc interval was longer in patients with PH than in controls [severe PH (mean PAP ≥ 60 mmHg): 428.6 ms ± 32.8, mild-to-moderate PH (mean PAP ≥ 25 mmHg and <60 mmHg): 423.1 ms ± 30.2; controls: 411.1 ms ± 28.4] [36], and in Rich's study, the QTc interval was 454.8 ms ± 29 in patients with PH compared with 429 ms ± 18 in controls [37]. The duration of the QTc interval in patients with nsVT (441 ms ± 34) or those who died (438 ms ± 25) during follow-up was longer than in patients with severe PH (428.6 ms ± 32.8) in Hong-Liang's study and showed values of QTc interval duration between the duration of patients with PH (454.8 ms ± 29) and controls (429 ms ± 18) in Rich's study. The mean QTc duration in our patients with nsVT who died during follow-up (*n* = 4) was 445 ms ± 45. The mean PAP of our patients (all patients, 40.8 mmHg) was lower than in the comparable group (patients with mild-to-moderate PH) in Hong-Liang's study (47.3 mmHg) and lower than in Rich's study (47.4 mmHg). In patients with nsVT, the values of mean PAP of Rich's, Hong-Liang's, and our patients were similar (about 47 mmHg). Compared with Rich's patients, PVR was lower in our patients (all patients, Table 3) but higher in patients with nsVT (835 versus 736 dyn s cm^−5^). Hong-Liang did not provide these data [36].

By extending Holter ECG monitoring time up to 72 h, the detection rate of patients with nsVT increased [10]. In three patients, nsVT was observed during the first 24 h, in seven patients between 24 and 48 h, and in two patients between 48 and 72 h of the recording. Additionally, as shown by Bass et al., prolongation of monitoring is useful and raises the number of detected arrhythmias [38].

On the whole, one-third of our patients with nsVT died during follow-up. Of course, the total number of patients in our trial is too low to draw statistically significant conclusions, but hemodynamic parameters and myocardial fibrosis, as discussed by Umar et al. and Rich et al. [3, 37], seem to be important for the development of arrhythmias in patients with PH.

The limitations of the current study consist in the moderate sample size (*n* = 78) and the low number of patients with nsVT (*n* = 12). Basically, our study was not designed to understand the molecular substrates or investigate the mechanisms initiating nsVT observed in patients with PH in our previous studies.

## 13. Conclusion

In patients with PH, nsVT occurs more often than previously reported, and patients with PH group 1 seem to be more at risk. Because of the moderate sample sizes, the prognostic relevance and risk factors for the development of nsVT in these patients cannot finally be assessed. Studies with a larger number of patients to obtain more information about the prognostic relevance of nsVT and risk factors (hemodynamic parameters and duration of QTc interval) for the development of nsVT are necessary. MRI can be useful for assessing myocardial fibrosis.

## Figures and Tables

**Table 1:** Baseline characteristics.

	Patients without nsVT	Patients with nsVT	Significance
	(*n* = 66)	(*n* = 12)	*p*
Diagnosis (PH group 1/4)	47/19	8/4	0.277
Age (years)	60 ± 14.7 (Median: 60.5)	66.6 ± 12.4 (Median: 66)	0.112
Gender (female/male)	50/16	8/4	0.277
PAH-specific medication (PDE5i/ERA/inh Prostanoid/CCB/Ima)	28/7/0/5/2	5/2/0/1/0	
Combination therapy (dual/triple)	PDE5i + ERA = 15,	PDE5i + ERA = 2,	
	PDE5i + inh Prostanoid = 3,	PDE5i + inh Prostanoid = 2	
	PDE5i + ERA + inh Prostanoid = 4,		
	PDE5i + Ima = 2		
Antiarrhythmic medication (BB/Digoxin)	11/1	1/0	
Angiotensin-converting enzyme inhibitors	14	3	
Coronary heart disease	8	2	0.274
Arterial hypertension	28	7	0.238
Diabetes mellitus	12	4	0.261
Cardiac pacemaker	0	1 (indication: bradyarrhythmia)	

BB: beta blocker; CCB: calcium channel blocker; ERA: endothelin receptor antagonist; Ima: imatinib; inh Prostanoid: inhaled Prostanoid; *n*: number; PDE5i: phosphodiesterase-5 inhibitor; PH: pulmonary hypertension; nsVT: nonsustained ventricular tachycardia.

**Table 2:** ECG parameters.

	Heart rate (beats/min)	*p*	QRS width (ms)	*p*	QT interval (ms)	*p*	Corrected QT interval (ms)	*p*
Survival								
yes (*n* = 57)	74 ± 13	0.823	93 ± 13	0.350	392 ± 31	0.503	432 ± 31	0.291
no (*n* = 21)	78 ± 14		92 ± 11		385 ± 36		438 ± 25	
Arrhythmia								
yes (*n* = 12)	76 ± 16	0.870	93 ± 8	0.195	393 ± 32	0.905	441 ± 34	0.470
no (*n* = 66)	74 ± 13		92 ± 13		389 ± 33		433 ± 29	

min: minute; ms: milliseconds; *n*: number; SD: standard deviation.

**Table 3:** Echocardiographic and hemodynamic data.

	All patients (*n* = 78)	Patients without nsVT (*n* = 66)	Patients with nsVT (*n* = 12)	*p* value
Echocardiography				
RA diameter (mm)	41.7	41.2	44.6	0.247
RV diameter (mm)	38.9	38.4	41.8	0.197
sPAP (mmHg)	63.3	61.6	72.7	0.163
TAPSE (mm)	20.1	20.9	15.7	0.001
Right heart catheterization				
mPAP (mmHg)	40.8	40.2	47.7	0.865
RAP (mmHg)	4.7	4.4	4.3	0.310
PCWP (mmHg)	7.9	7.8	7.2	0.688
PVR (dyn·s·cm^−5^)	563	532	835	0.353
CI (l/min/m^2^)	2.73	2.81	2.42	0.390
ven sO_2_ (%)	67	68	64	0.700

CI: cardiac index; cm: centimeter; dyn: Dyne; Hg: mercury; l: liter; m: meter; mm: millimeter; mPAP: mean pulmonary arterial pressure; *n*: number; nsVT: nonsustained ventricular tachycardia; PCWP: pulmonary capillary wedge pressure; mPAP: mean pulmonary arterial pressure; PVR: pulmonary vascular resistance; RA: right atrium; RAP: right atrial pressure; RV: right ventricle; s: second; sPAP: systolic pulmonary arterial pressure; TAPSE: tricuspid annular plane systolic excursion; ven sO_2_: venous oxygen saturation.
